# The ‘Relevant’ Stability of Proteins with Equilibrium Intermediates

**DOI:** 10.1100/tsw.2002.196

**Published:** 2002-05-04

**Authors:** Javier Sancho, Marta Bueno, Luis A. Campos, Juan Fernandez-Recio, Maria Pilar Iran, Jon Lopez, Claudio Machicado, Idolka Pedroso, Miguel Toja

**Affiliations:** Departamento de Bioquímica y Biología Molecular y Celular, Facultad de Ciencias, Universidad de Zaragoza, 50009-Zaragoza, Spain; The Scripps Research Institute, Molecular Biology TPC-28, 10550 North Torrey Pines Road, La Jolla, CA 9203, USA; Operón S.A., Camino del Plano 19, Cuarte de Huerva, 50410-Zaragoza, Spain

**Keywords:** protein engineering, protein folding, protein intermediate, protein stabilisation, protein stability, three-state

## Abstract

Proteins perform many useful molecular tasks, and their biotechnological use continues to increase. As protein activity requires a stable native conformation, protein stabilisation is a major scientific and practical issue. Towards that end, many successful protein stabilisation strategies have been devised in recent years. In most cases, model proteins with a two-state folding equilibrium have been used to study and demonstrate protein stabilisation. Many proteins, however, display more complex folding equilibria where stable intermediates accumulate. Stabilising these proteins requires specifically stabilising the native state relative to the intermediates, as these are expected to lack activity. Here we discuss how to investigate the ‘relevant’ stability of proteins with equilibrium intermediates and propose a way to dissect the contribution of side chain interactions to the overall stability into the ‘relevant’ and ‘nonrelevant’ terms. Examples of this analysis performed on apoflavodoxin and in a single-chain mini antibody are presented.

## STABILISATION OF PROTEINS WITH A TWO-STATE EQUILIBRIUM

The conformational stability of a protein is the free energy difference of the native/denatured equilibrium.

$$N \xleftrightarrow{\Delta G} D$$

Where no intermediates complicate this equilibrium, the stability can be easily measured from thermal or chemical denaturation[1]. Fuelled by the interest in protein stability, small model proteins have been used to investigate both the principles and practical strategies of protein stabilisation[2,3]. Although some basic questions regarding what stabilises proteins may not be settled[4,5] there are now various ways to attempt, with a reasonable probability of success, the increase of protein stability from a judicious analysis of protein structure[6]. The question is, Are these strategies similarly useful to stabilise proteins with more complex equilibria?

## THE 'RELEVANT' CONFORMATIONAL STABILITY OF PROTEINS WITH COMPLEX EQUILIBRIA: THE THREE-STATE CASE

Let us consider a simple three-state folding equilibrium with a single intermediate conformation appearing at mildly denaturing conditions (e.g., moderate urea concentration or moderately high temperatures) before the full denaturation takes place.

$$N \xleftrightarrow{\Delta G_{NI}} I \xleftrightarrow{\Delta G_{ID}} D$$

For proteins of this kind, the conformational stability is made of two terms that, respectively, represent the stability of the native conformation relative to the intermediate ($\Delta G_{NI}$) and that of the intermediate relative to the denatured state ($\Delta G_{ID}$). However, provided the intermediate is no longer active (and the odds are it will not be), the 'relevant' conformational stability is given by just the first term, $\Delta G_{NI}$. The point is that the energetics of NI equilibria are so poorly understood that it is not clear whether the strategies found to stabilise two-state proteins will work well for proteins with equilibrium intermediates. If the energetics of protein intermediates are close to those of denatured states, there is no problem; but if, energetically, intermediates are not very different from the native state[7], then we face a difficult problem, because anything that stabilises the native conformation will similarly stabilise the intermediate, and the 'relevant' stability will not change much.

## HOW TO DISTINGUISH BETWEEN TWO-STATE AND THREE-STATE EQUILIBRIA

The first thing to do when working with a particular protein is to clarify whether its equilibrium is two-state or more complex. Sometimes the cooperativity of an unfolding transition (chemical or thermal) is misleadingly invoked as proof of two-state behaviour. In fact, unfolding spectroscopic data of complex equilibria can be, at first sight, indistinguishable from those of simple equilibria. Differential scanning calorimetry offers a criterion of two-state behaviour in the equivalence of the van't Hoff and calorimetric enthalpies, but in some cases it can fail to detect intermediates[8,9]. A different and useful two-state criterion is the so-called superposition test[10]. Proteins with simple equilibria must display the same half denaturant concentration or melting temperature regardless of the spectroscopic technique used to monitor the unfolding. When unfolding curves followed by, say, fluorescence and circular dichroism (CD) cannot be superimposed, then an equilibrium intermediate must be present.

## ENERGETICS OF THE NI EQUILIBRIUM: f -ANALYSIS

Inspired by the $\phi$-analysis of the structure of transition states and of transient folding intermediates[11] we have recently proposed a way to investigate the integrity in equilibrium intermediates of side-chain interactions present in the native state[9]. The procedure is as follows: First, the unfolding transition of a given protein is monitored using several spectroscopic techniques. We use fluorescence, far-UV CD, near-UV CD, and near-UV absorbance unfolding curves, which are globally analysed to get the thermodynamic parameters of the NI and ID equilibria. Then mutant proteins, in which an interaction present in the native wild-type structure is broken, are constructed and analysed in the same way as the wild-type protein. From the shifts in the $T_m$s of the NI and ID equilibria, the free energy changes introduced by the mutation are calculated. Finally, a $\phi_I$ parameter is defined as

$$\phi_I = \Delta\Delta G_{ID}/\Delta\Delta G_{ND}$$

When $\phi_I = 1$, the broken interaction is as present in the intermediate as it is in the native state, and when $\phi_I$ is 0, then the interaction is absent in the intermediate (Fig. 1). Other $\phi_I$ values, between 0 and 1, might in principle reflect a variety of intermediate conformations, but if the unfolding of the intermediate is cooperative (as is often the case and which can be assessed from the shape of the unfolding transition), they represent a partial formation of the probed interaction.

This analysis can be used to study both stabilising and destabilising single mutations and, indeed, it can also be used to study double mutations involving pairs of interacting residues. The point is that, by this procedure, we can estimate the degree of formation of a given interaction in the intermediate. Four extreme cases are possible:

Ia: The interaction probed is protein stabilising and it is as formed in the intermediate as in the native conformation. Removing the interaction will not change the ‘relevant’ stability. Similarly, the introduction of one new such interaction will not change the ‘relevant’ stability either.

Ib: The interaction is protein stabilising and it is not formed in the intermediate. Removing the interaction will decrease the ‘relevant’ stability, while engineering one new such interaction will increase the ‘relevant’ stability.

IIa: The interaction is protein destabilising (for example, an electrostatic repulsion between two neighbouring charges) and it is as formed in the intermediate as in the native conformation. Removing the interaction will not change the ‘relevant’ stability.

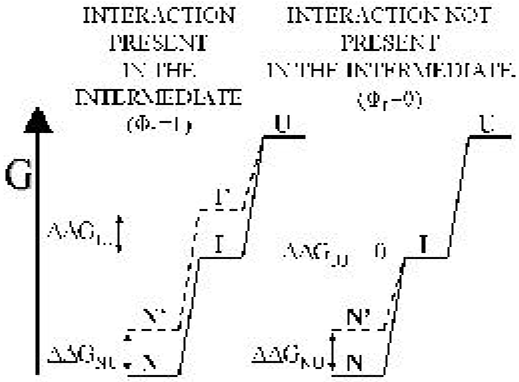

**FIGURE 1**. Energy diagram showing possible effects of mutations on the energetics of the two equilibria (NI and ID) and their relationship to the integrity parameter $\phi_I$.

IIb: The interaction is protein destabilising and it is not formed in the intermediate. Removing the interaction will increase the ‘relevant’ stability, while engineering one new such interaction will decrease it.

The key is to know the types of engineerable interactions that can stabilise proteins (which is, to some extent, known from the analysis of two-state proteins) and to know, or at least to be able to anticipate, what interactions of those present in native conformations tend to be retained in protein intermediates. To contribute to the clarification of these issues, we are currently investigating in our laboratory side-chain interactions in three-state and four-state equilibrium proteins. A few examples follow.

## HYDROGEN BONDS AND HYDROPHOBIC INTERACTIONS IN THE FLAVODOXIN THERMAL INTERMEDIATE

Apoflavodoxin is a well-characterised model protein that displays a two-state unfolding equilibrium behaviour towards chemical denaturation but that, in the thermal denaturation, populates an equilibrium intermediate[6,8,9,12].

The persistence of surface hydrogen bonds in the thermal intermediate has been investigated to obtain structural information (Fig. 2). All hydrogen bonds analysed are present although debilitated in the intermediate[9].

If this result can be extrapolated to other intermediates, and assuming hydrogen bonds stabilise proteins, our analysis indicates that the overall stabilisation of the native state relative to the dena-

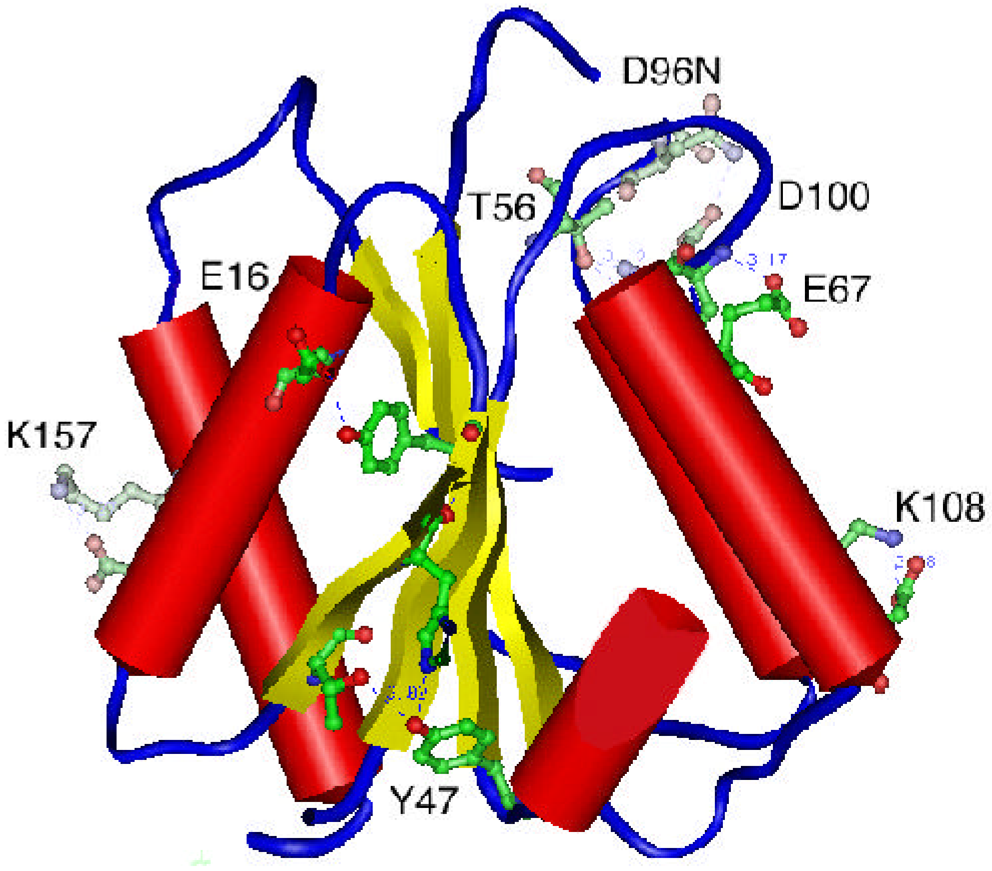

**FIGURE 2.** Ribbon diagram of apoflavodoxin secondary structure showing mutated hydrogen bonds.

tured one afforded by engineering new hydrogen bonds into proteins with intermediates would be only partly realised in terms of ‘relevant’ stability. For this type of interaction, however, the insight is complicated by the fact that the net contribution of hydrogen bonds to protein stability is still debated[13].

The contribution of hydrophobic interactions to protein stability is more widely accepted. The persistence of hydrophobic interactions involving core residues in the apoflavodoxin thermal intermediate is currently under investigation. Our preliminary analysis of a cavity-creating mutant (L50A, Fig. 3) indicates that the overall interaction of a core leucine (van der Waals plus hydrophobic effect) is retained, to around 60% of its value, in the intermediate.

We can thus speculate, extrapolating this result, that filling holes in proteins displaying equilibrium intermediates can increase the ‘relevant’ stability, although to a lower extent (around 40%) than in the case of two-state equilibrium proteins. This stabilisation can nevertheless be substantial.

## ELECTROSTATIC INTERACTIONS IN THE scFV INTERMEDIATE

Electrostatic interactions can stabilise or destabilise proteins according to their number and distribution around the protein molecule[14]. We are investigating the persistence of electrostatic interactions in protein intermediates using a single-chain Fv antibody fragment (scFv). Our analysis indicates that its equilibrium unfolding is four state (submitted). We have performed a global analysis of the unfolding equilibrium at different pH values and used the data to calculate a $\phi$-like parameter that accounts for the persistence of electrostatic interactions (Fig. 4) in the more stable of the two identified scFv intermediates.

According to our data, electrostatic interactions are partly retained in this intermediate to about 40%. Thus it seems possible to increase the ‘relevant’ stability of this scFv by improving the electrostatic interactions present in the native state. The fact that scFv molecules contain two folding do-

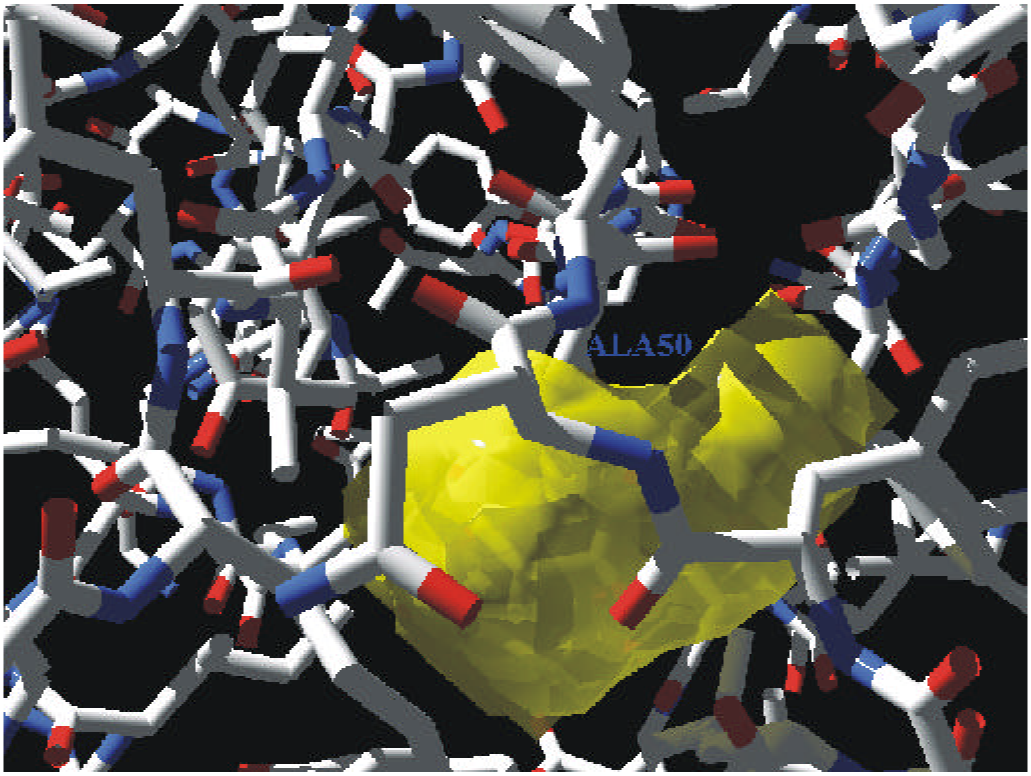

**FIGURE 3.** Model of the cavity created in wild-type apoflavodoxin by replacing L50 with Ala.

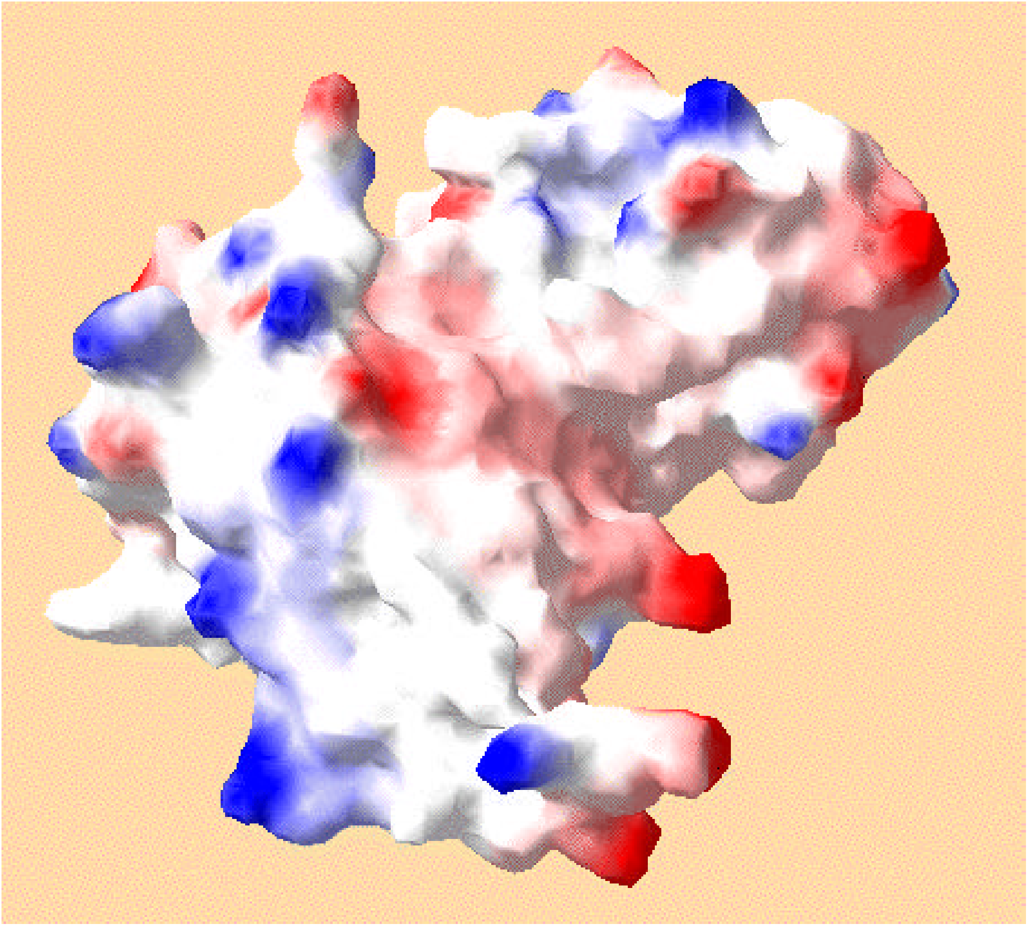

**FIGURE 4.** Electrostatic potential surface of an anti-HbsAg scFv fragment. Modelled from de 4fab and 1sbs using InsightII. Surface calculated with SpdbViewer.

mains, however, gives rise to alternative explanations of this fractional electrostatic ϕ-value, and the feasibility of using electrostatic interactions to increase the ‘relevant’ conformational stability of proteins with intermediates remains to be tested in simpler one-domain proteins.

## PROSPECTS

As more and more complex proteins are being studied and utilised, the need for an understanding of the energetic differences of native and intermediate conformations will increase. This will certainly be stimulated by the mounting evidence that some diseases may be related to the accumulation of protein intermediates or to a reduced stability of the native state caused by mutations[15]. In particular, any recovery of misfolded proteins showing intermediates will have to concentrate on exploiting structural and energetic differences between native and intermediate states. Although it is true that two-state equilibrium proteins are the best models for accurately quantifying protein energetics, it is also clear that much effort must be devoted to delineate and, if possible, generalise the differences between native proteins and intermediate states. To that end, the analysis here proposed can help decide the type of interactions best suited to increase the ‘relevant’ conformational stability of proteins.

## ACKNOWLEDGEMENTS

Financial support from grants 2FD1997-0758 (FEDER+DGES, Spain), BMC2001-2522 (DGI, Spain), and P120/2001 (DGA, Spain) is acknowledged.
